# KIDMED 2.0, An update of the KIDMED questionnaire: Evaluation of the psychometric properties in youth

**DOI:** 10.3389/fnut.2022.945721

**Published:** 2022-11-08

**Authors:** Miguel A. López-Gajardo, Francisco M. Leo, Pedro Antonio Sánchez-Miguel, Dori López-Gajardo, Candelaria Soulas, Miguel A. Tapia-Serrano

**Affiliations:** ^1^Faculty of Sport Sciences, University of Extremadura, Cáceres, Spain; ^2^Faculty of Teacher Training, University of Extremadura, Cáceres, Spain; ^3^Cliniber, Diet Therapy and Nutritional Education, Badajoz, Spain; ^4^Nut&Cook, Madrid, Spain

**Keywords:** dietary pattern, eating behavior, KIDMED, Mediterranean diet, youths

## Abstract

**Background and aims:**

As children and adolescents' eating patterns have changed over the last few years, researchers have found inconsistencies in the current questionnaires. Therefore, this research aims to (i) update the 2019 KIDMED questionnaire; and (ii) test the psychometric properties of this new questionnaire.

**Method:**

A study with 419 children and adolescents in southwestern Spain was conducted in 2021. The new version of the KIDMED 2.0 was tested, which measures adherence to the Mediterranean diet through 16 items, of which 12 are positive, and 4 are negative. Content validation involved consultation with nutritionists, experts, and adolescents to assess whether the questionnaire was reliable and valid regarding dietary patterns associated with the Mediterranean diet. The expert assessment provided content validity indices for the clarity and representativeness of the questionnaire. Construct validity and test-retest reliability involved 419 students (*M*_age_ = 14.40 ± 2.00) from southwestern Spain. Students responded twice (one week apart) to the KIDMED developed in the previous stage and completed a 7-day dietary record.

**Results:**

Regarding validity, results show a moderate agreement for 10 items (ranging between 0.21 and 0.47) of the KIDMED and the 7-day dietary record. Concerning Items 3, 4, 5, and 6, the agreement was slight (ranging between 0.08 and 0.17), whereas the agreement for Item 8 was low. Cohen's kappa showed that most items had moderate to substantial test-retest reliability. Also, kappa showed significant test-retest values for all items (*p* < 0.001).

**Conclusion:**

The new version of the KIDMED 2.0 was shown to be a reliable and valid instrument to measure adherence to the Mediterranean diet in children and adolescents.

## Introduction

Mediterranean diet is a dietary pattern characterized by the intake of a large number of vegetables, fruits, whole-grain bread, whole-grain rice, pulses and nuts, moderate amounts of fish and dairy products (especially, cheese and yogurt), while limiting the consumption of red meat and using virgin olive oil as the main source of fat (1). Moreover, the Mediterranean diet recommendations encourage reducing as much as possible the consumption of ultra-processed foods, soft drinks, sweets, alcohol, and tobacco (2). This diet is particularly notable for the consumption of fresh and local foods, culinary activities, and food variety (especially fruit and vegetables), which are a fundamental part of these eating habits (3). However, urbanization, the integration of women into the labor market, retail development, and economic globalization have caused considerable changes in the eating habits of the Mediterranean population, especially in children and adolescents (3–5). Overconsumption of foods high in sugar (e.g., soft drinks, sweets, fruit juice, pastries), and refined (cereals or refined grains) or ultra-processed foods have replaced traditional and local Mediterranean foods, generating negative effects on the health of young people (4, 5).

In this sense, in recent years, there has been great interest in improving these habits, due to the worrisome levels of obesity in different populations, with the childhood stage being highlighted (6). In addition, the prevalence of pre-obesity or obesity is significantly higher in Iberian countries such as Spain, where rates are over 30% in children (7). To address this problem, the Mediterranean diet has been one of the key references to establish adequate eating patterns, select the foods that we should consume or try to avoid, or even determine the way to cook these foods (3). To assess the extent to which people eat a healthy diet and follow the patterns of the Mediterranean diet, a daily record of the population's food consumption would have to be developed. However, the daily registry shows considerable difficulty in assessing these dietary habits in large populations because it requires the participants' commitment over a long period of time.

To address this limitation, Serra-Majem et al. (8) designed a questionnaire that represents adherence to the Mediterranean diet cross-sectionally and over a short period of time. Specifically, this questionnaire, the KIDMED, consists of 16 questions and assesses the frequency of consumption of different foods, eating breakfast, eating fast food, etc. (9). Since the publication of the KIDMED questionnaire (8), this instrument has been extensively used to measure adherence to the Mediterranean diet in children and adolescents (3, 10). Given that it is a simple instrument to use, many countries in the Mediterranean area such as Croatia (11), Greece (12–14), Hungary (10), Israel (15), Italy (16–18), Lebanon (19), Lithuania (11), Portugal (20–22), Serbia (11), Spain (23–26), and Turkey (27–29) have used this instrument to measure the adherence to the Mediterranean diet in children and young people. Furthermore, other countries which, due to their geographical position and gastronomic culture, have access to foods typical of the Mediterranean pattern such as Brazil (30), Chile (31), Colombia (32), Cyprus (33), Estonia (34, 35), Finland (36), and the United States (37–39) have also used the questionnaire.

These versions of the KIDMED were a breakthrough in the assessment of adherence to the Mediterranean diet. However, few studies have investigated the psychometric properties of the KIDMED questionnaire (9). The study conducted by Aparicio-Ugarriza et al. (10) analyses the psychometric properties of the KIDMED, but they only consider the index score. Therefore, to our knowledge, there are only three studies that have examined the psychometric properties of the KIDMED in children and adolescents in two countries: Colombia (32), Brazil (30), and Portugal (21). Specifically, the study conducted by Carrillo and Ramírez-Vélez (32) showed adequate overall internal consistency of the questionnaire, except for Items 1, and 2. Moreover, neither of these studies took into account the updating of the KIDMED items performed by Altavilla and Caballero-Perez (5), which could justify the inconsistencies found in the two studies. As the description of some of the KIDMED items was too general and could lead to internal inconsistencies with the Mediterranean diet, such as consumption of juices or nonwhole-grain pasta and rice, Altavilla and Caballero-Perez (5) proposed a modification and update of Item 1 (i.e., replace “Drinks a fruit juice every day” with “Eats a fruit every day”), Item 8 (i.e., replace “Pasta or rice almost daily” with “Consumes whole-grain pasta or whole-grain rice almost every day”), Item 9 (i.e., replace “Cereal or cereal product for breakfast” with “Eats whole cereals or whole-grains for breakfast”) and Item 12 (i.e., replace “No breakfast” with “Skips breakfast”) of the KIDMED questionnaire to improve the assessment of adherence to the Mediterranean diet in children and adolescents. However, in this new version, the validity and reliability to support the proposed changes to the KIDMED questionnaire were not examined.

Furthermore, the 2019 KIDMED update did not include some important aspects of the Mediterranean diet, such as the consumption of ultra-processed foods and the way of consuming food (i.e., cooking activities), which are fundamental aspects to assess adherence to the Mediterranean pattern (3). Therefore, because previous studies have not tested the validity and reliability of the updated instrument and because there are still items that need to be revised to fit the Mediterranean diet, this research aims to (i) update the 2019 KIDMED questionnaire; and (ii) test the psychometric properties, in terms of reliability and validity, of the updated KIDMED 2.0 in a group of Spanish children and adolescents.

## Methods

This study consisted of two phases. Phase I was used for content validity (performing a total of four sub-phases), and Phase II was used to measure the reliability (using techniques of test-retest) and construct validity of the KIDMED 2.0 questionnaire. This research was approved by the Bioethics Committee of the University of the principal author (120/2018). All participants were treated according to the ethical guidelines provided by the American Psychological Association (2019).

### Phase I: Content validity

#### Phase Ia: Initial development and item selection of the KIDMED questionnaire

First, the authors (i.e., the first, second, third, and sixth authors of the present study) reviewed the items included in previous adaptations and validations of the KIDMED questionnaire (8, 21, 29, 31, 32). Second, they conducted an initial pool of 16 items to assess adherence to the Mediterranean diet based on the previous questionnaire of Altavilla and Caballero-Perez (5).

Each researcher independently evaluated the content of the 16 KIDMED items. Subsequently, independently proposed improvements or modifications were discussed among all members of the research team. To maintain or modify any item of the questionnaire, 75% agreement among the research team members was required. Finally, maintaining the structure of the original KIDMED, the authors agreed on an initial questionnaire comprised of 16 items based on the assessment of eating habits, of which 12 denote positive connotations and 4, negative aspects (see Supplementary Table 1). The authors' group was composed of university professors with extensive experience in the investigation of healthy habits and validation of questionnaires, with experience in the creation and adaptation of methodological scales. All these steps were conducted to obtain information on each item's clarity, relevance, and specificity (40, 41).

#### Phase Ib: Panel of experts

The first item pool was sent to two expert nutritionists in the Mediterranean diet (i.e., the fourth and fifth authors of the present study). Each nutritionist reviewed all the items to increase their precision according to the Mediterranean diet recommendations and correct drafting problems. The nutritionists reported potential problems with the daily consumption of pre-cooked food, the intake of fruits and vegetables per day, the quality of the cereals and olive oil, the characteristics of breakfast, cooking activities, or the number of industrial pastries ingested. For example, the item “Does not eat breakfast” (see Supplementary Table 1) being valued negatively with a−1 was changed to “When I eat breakfast, I eat pastries, cookies, juices, smoothies or processed products” (see Supplementary Table 2).

Afterward, the proposed changes and/or modifications were discussed through a think-aloud protocol between the team of nutritionists and the members of the research team. Two meetings were held until 100% agreement was reached between the team of nutritionists and the research team. The result of this process was a second version of the questionnaire, which maintained the structure of the original KIDMED (i.e., 16 questions, of which 12 denoted positive connotations and 4 denoted negative connotations; see Supplementary Table 2).

#### Phase Ic: External panel of expert nutritionists

To review the second item pool, a total of three meetings (two online and one face-to-face) were carried out through a think-aloud protocol between an external expert group of six nutritionists and the two nutritionists who participated in the previous step. All versions were discussed and merged into one, in line with the consensual approach. In addition, all members of this expert group of nutritionists had more than 8 years of experience (9.40 ± 2.10) in the nutrition of children and adolescents and adherence to the Mediterranean diet. Therefore, these three research actions independently incorporated the opinions of experts in the field to improve the content validity of the KIDMED 2.0 Questionnaire.

Subsequently, the nutritionists' responses were evaluated to modify the problem items. In general, qualitative comments were included so the participants could understand the words contained in several items (e.g., Items 3, 7, or 13, see Supplementary Table 3). Also, we modified the consumption per day or week in Item 12 (see Supplementary Table 3).

#### Phase Id: Item comprehension and final panel of experts

To know whether participants optimally interpret each item is essential to test the content validity. In this regard, to know the item comprehension and after obtaining ethical approval, we distributed the questionnaire to 9 children (*n* = 7) and adolescents (*n* = 2) of both sexes (12.22 ± 2.46; 45.55% girls). Participants were purposively sampled, as we sought to recruit information-rich cases (42). The inclusion criterion stipulated that only children or adolescents from southwestern Spain could participate. Moreover, adopting a maximum variation sampling approach (43), children and adolescents between 9 to 17 years were sampled. All authors identified potentially eligible participants *via* personal friendships, contacting them to inform them about the study and inviting them to participate. Participants provided verbal informed consent before participation. Then, a single semi-structured interview was conducted with each participant to gain insight into how participants could understand and respond to the KIDMED 2.0 Questionnaire. Interviews were conducted by the authors, in the Spanish language (i.e., the first language of all the authors and participants), online, and at a mutually convenient time. The authors asked the participants to read all the questions and to verbalize only those items they did not understand. The authors collected the children and adolescents' qualitative comments on the items to identify problematic items. Finally, Items 8 (i.e., methods of cooking) and 11 (i.e., the characteristics of olive oil) were the only ones identified as problematic. For instance, referring to Item 8, one participant indicated: “I don't know how my mother cooks and I don't know the difference between fried or grilled.” Regarding Item 11, some students admitted that they could not identify olive oil: “I know that my mother normally uses oil, but I don't understand the difference between these two types of oil.” Finally, all members (i.e., authors and participants) determined that these drawbacks could be solved by modifying the items. For example, they added the amount of oil used for cooking in Item 8 or the color of the oil in Item 11 (see Supplementary Table 4).

### Phase II: Field testing

#### Design and participants

In this longitudinal study, intentional cluster sampling was used for sample selection. The geographical proximity to the schools and the researchers' possibilities to access the sample were considered. The inclusion criterion for the investigation stipulated that participants had to be children and adolescents. Moreover, the research team specified that students could participate if they completed the first and second assessments of KIDMED 2.0 and the 7-day DR. The data were collected from March 2021 to June 2021 in southwestern Spain. Based on the eligibility criteria, a final sample of 419 students, aged 9 to 17 years (14.40 ± 2.00), of whom 208 were children (12.13 ± 0.90, 53.84% girls) and 211 were adolescents (15.45 ± 1.04, 57.81% girls), participated in the investigation. The minimum sample size required for the study was 375 youths. It has been shown that a sample of at least 200 respondents is necessary to achieve temporal stability (44).

#### Measures

##### Adherence to the mediterranean diet

The version of the KIDMED 2.0 developed in Phase I was used to measure adherence to the Mediterranean diet in children and young people. This tool comprises 16 questions based on the assessment of eating habits, of which 12 denote positive connotations and 4, negative aspects. The index of adherence to the Mediterranean diet was calculated as the sum of each answer and ranged from −4 to 12.

##### Daily record (7-day dietary record)

To assess children's and adolescents' daily record of adherence to the Mediterranean diet, we used a 7-day dietary record (DR). The 7-day DR record has been recognized as the gold standard of the dietary assessment methods and is used as a reference in calibration or validation studies that employ other less rigorous and less expensive methods (44). On days 1 through 7, participants completed at home a daily record to report their food and beverage intake for 5 weekdays and 2 days on the weekend. Emphasis was placed on providing comprehensive information relative to all foods eaten.

#### Procedure

**Figure 1:**
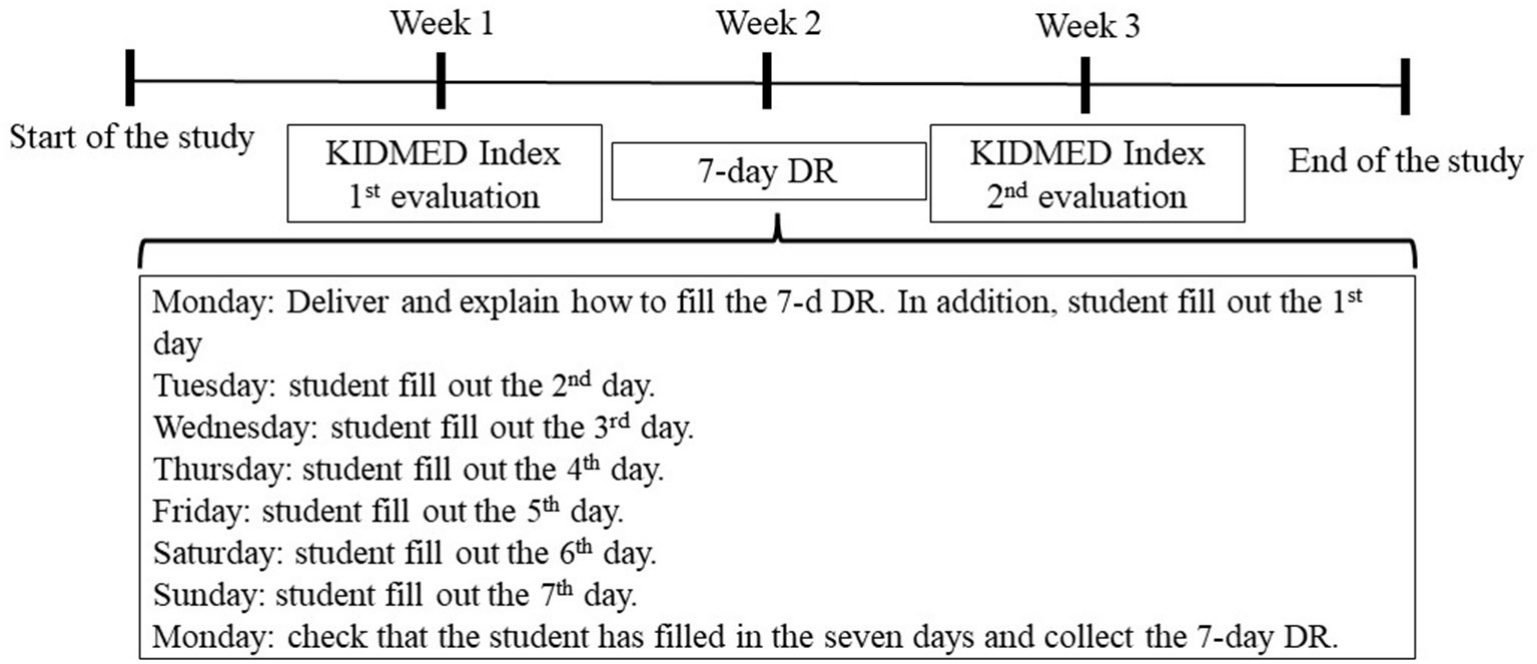
Data gathering timeline. KIDMED Index; DR, Dietary-Record.

The research team contacted the principal and Physical Education teachers at each school to conduct this study. Parents were informed of the nature and purpose of the study by letter. Written informed consent was required both from the participants and their parents/legal guardians. The paper-and-pencil questionnaire was administered in a Physical Education lesson by one member of the research team. The first assessment of adherence to the Mediterranean diet was carried out using the version of the KIDMED 2.0 questionnaire for children and adolescents. The time to complete the survey was 20 min. The children and adolescents then took home the 7-day DR to report all the food they ate during the next week (i.e., Monday to Sunday). Previously, the research team gived participants several advises to write down the foods they ate during the this week. They also reminded the participants that they did not need to include the size of the foods, only the name and the quantities. An example of the daily record was also provided. Therefore, students estimated food intake using food names and a portion-size guide (45). Two weeks after the first assessment of KIDMED 2.0, the second assessment of adherence to the Mediterranean diet was conducted using the version of the KIDMED 2.0 for children and adolescents (Figure 1). In line with previous studies (10, 21, 46), a period of 2 weeks is considered sufficient to eliminate the influence of the responses of the first test on the results of the retest. One of the research team members was also present during the assessment.

### Data analysis

The SPSS Statistics v.23.0 software was used for data analysis. Cohen's Kappa with a 95% confidence interval between test and retest responses was used to examine the test-retest reliability of the items. Cohen's Kappa with a 95% confidence interval between responses in the test and the 7-day DR was used to examine the construct validity of the items. The descriptive terms of Landis and Koch (1) were used to characterize values of kappa: < 0%, poor; 0–20%, slight; 21–40%, fair; 41–60%, moderate; 61–80%, substantial; and 81–100%, almost perfect.

## Results

### Test-retest reliability

Descriptive statistics of the test-retest reliability of the KIDMED 2.0 questionnaire are presented in Table 1. Overall, Cohen's kappa coefficients for the 16 items of the KIDMED 2.0 showed that most of items had moderate to substantial test-retest reliability (47). Also, significant test-retest kappa statistic values were found for all items (*p* < 0.001). However, for Item 3, regarding protein consumption, and Item 8, which refers to how food is cooked at home, the agreement was fair (κ = 0.36 and 0.29, respectively). The KIDMED index score had a moderate test-retest reliability (κ = 0.48) and a significant kappa statistic value (*p* < 0.001). Finally, there was little variation in Cohen's kappa coefficients in the results for all the items the KIDMED 2.0 by age (see Supplementary Table 5) and sex (see Supplementary Table 6) as well as for the KIDMED 2.0 index score.

**Table 1:** Test-retest and reliability values of the items of the KIDMED 2.0.

**KIDMED 2.0**	**Baseline Test**		**After 2 weeks Re-Test**		**Cohen's Kappa**	**95% CI**
	**Yes (%)**	**No (%)**	**Yes (%)**	**No (%)**		
1. I eat two or more servings of fruit a day	214 (51.2)	204 (48.8)	210 (50.2)	208 (49.8)	0.64^***^	0.56–0.71
2. I eat one or more servings of vegetables and/or raw or cooked vegetables per day	195 (46.4)	224 (53.6)	182 (43.5)	236 (56.5)	0.51^***^	0.42–0.59
3. I eat one portion at lunch and another at dinner of legumes, meat (chicken, turkey or rabbit, or other lean meats), fish and/or eggs a day	343 (82.1)	75 (17.9)	315 (75.4)	103 (24.6)	0.36^***^	0.26–0.47
4. More than half of the food I eat is of plant origin (fruits, vegetables, legumes, nuts, potatoes, whole grains)	266 (63.6)	152 (36.4)	247 (59.1)	171 (40.9)	0.41^***^	0.31–0.49
5. When I eat lean meat, eggs and/or fish, they are usually fresh and minimally processed	331 (79.2)	87 (20.8)	340 (81.3)	78 (18.7)	0.49^***^	0.39–0.60
6. I eat precooked food or fast-food such as pizzas and hamburgers one or more times a week	225 (53.8)	193 (46.2)	241 (57.7)	177 (42.3)	0.53^***^	0.44–0.61
7. I eat three or more servings of legumes (chickpeas, white beans, lentils, peas) a week	371 (88.8)	47 (11.2)	358 (85.6)	60 (14.4)	0.54^***^	0.42–0.66
8. At home, food is usually cooked in the oven, grilled (a frying pan with little oil) or boiled (do not using a fryer)	364 (87.3)	53 (12.7)	364 (87.3)	53 (12.7)	0.29^***^	0.16–0.42
9. When I eat cereals (pasta, rice, quinoa, couscous), I always eat whole grains	96 (23.0)	322 (77.0)	121 (28.9)	297 (71.1)	0.49^***^	0.39–0.58
10. I eat a serving of natural or roasted nuts without salt at least 3 times a week	101 (24.2)	316 (75.8)	116 (27.8)	301 (72.2)	0.53^***^	0.44–0.63
11. At home, virgin olive oil (dark green) is used instead of sunflower oil (yellow)	361 (86.4)	57 (13.6)	360 (86.1)	58 (13.9)	0.51^***^	0.39–0.63
12. I drink commercial soft drinks, juices and/or shakes one or more times during the week	293 (70.1)	125 (29.9)	276 (66.0)	142 (34.0)	0.60^***^	0.52–0.68
13. When I eat a portion of dairy products, they are always natural (milk, yogurt without sugar, fresh cheese) or minimally processed	300 (71.8)	119 (28.2)	309 (73.9)	109 (26.1)	57^***^	0.48–0.66
14. When I have breakfast, I eat pastries, cookies, juices, smoothies or processed products	243 (58.4)	174 (41.6)	253 (60.5)	165 (39.5)	0.63^***^	0.55–0.70
15. When I eat breakfast, I eat unprocessed or minimally processed foods (fruit, nuts, eggs, or whole wheat bread)	133 (31.8)	286 (68.2)	136 (32.5)	282 (67.5)	0.56^***^	0.47–0.64
16. I eat industrial pastries (sweets, cookies, snacks, or chocolate) and sweets (crisps, worms, candies, or jellies) several times a week	271 (64.8)	147 (35.2)	289 (69.1)	129 (30.9)	0.72^***^	0.47–0.64
**KIDMED 2.0 index score**						
Poor ( ≤ 3)	127 (30.3)		144 (34.4)		0.48^***^	0.41–0.55
Average (4–7)	223 (53.2)		197 (47.0)			
Good (≥8)	69 (16.5)		78 (18.6)			

Significant results:

*** p < 0.001.

### Construct validity

Descriptive statistics of the KIDMED 2.0 questionnaire and the 7-day DR are presented in Table 2. Results show a moderate agreement for 10 items (ranging between 0.21 and 0.47) between the items of the KIDMED 2.0 and the 7-day DR. Furthermore, concerning Items 3, 4, 5, and 6, related to the consumption of protein, plant-based foods, fresh and minimally processed foods, and pre-cooked or fast-food, the agreement was slight [ranging between 0.08 and 0.17 (47)]. However, for Item 8, regarding how food is cooked at home, and Item 11, about the type of olive oil used, the agreement was poor. Significant kappa statistic values were found for all items (*p* < 0.001), except for Items 3, 8, and 11. The KIDMED index score had weak construct validity (κ = 0.17), and a significant kappa statistic value (*p* < 0.001). Lastly, the construct validity results of the KIDMED 2.0 by age (see Supplementary Table 7) and sex (see Supplementary Table 8) showed similar values for Cohen's kappa coefficients for all items.

**Table 2:** KIDMED 2.0 and 7-day dietary record values of the items.

**KIDMED 2.0**	**Baseline Test**		**7-day Dietary Record**		**Cohen's Kappa**	**95% CI**
	**Yes (%)**	**No (%)**	**Yes (%)**	**No (%)**		
1. I eat two or more servings of fruit a day	212 (51.7)	198 (48.3)	208 (50.7)	202 (49.3)	0.37^***^	0.28–0.46
2. I eat one or more servings of vegetables and/or raw or cooked vegetables per day	194 (47.3)	216 (52.7)	147 (35.9)	263 (64.1)	0.27^***^	0.18–0.36
3. I eat one portion at lunch and another at dinner of legumes, meat (chicken, turkey or rabbit, or other lean meats), fish and/or eggs a day	337 (82.2)	73 (17.8)	208 (50.7)	202 (49.3)	0.08	0.00–0.15
4. More than half of the food I eat is of plant origin (fruits, vegetables, legumes, nuts, potatoes, whole grains)	261 (63.7)	149 (36.3)	185 (45.1)	225 (54.9)	0.10^*^	0.01–0.19
5. When I eat lean meat, eggs and/or fish, they are usually fresh and minimally processed	326 (79.5)	84 (20.5)	278 (67.8)	132 (32.2)	0.14^**^	0.03–0.24
6. I eat precooked food or fast-food such as pizzas and hamburgers one or more times a week	220 (53.7)	190 (46.3)	325 (79.3)	85 (20.7)	0.17^***^	0.09–0.25
7. I eat three or more servings of legumes (chickpeas, white beans, lentils, peas) a week	364 (88.8)	46 (11.2)	336 (82.0)	74 (18.0)	0.32^***^	0.20–0.44
8. At home, food is usually cooked in the oven, grilled (a frying pan with little oil) or boiled (do not using a fryer)	59 (88.1)	8 (11.9)	42 (62.7)	25 (37.3)	−0.07	−0.25–0.10
9. When I eat cereals (pasta, rice, quinoa, couscous), I always eat whole grains	94 (22.9)	316 (77.1)	65 (15.9)	345 (84.1)	0.27^***^	0.16–0.37
10. I eat a serving of natural or roasted nuts without salt at least 3 times a week	101 (24.8)	307 (75.2)	55 (13.5)	353 (86.5)	0.21^***^	0.30–0.51
11. At home, virgin olive oil (dark green) is used instead of sunflower oil (yellow)	46 (88.5)	6 (11.5)	45 (86.5)	7 (13.5)	−0.14	−0.22–0.06
12. I drink commercial soft drinks, juices and/or shakes one or more times during the week	290 (70.7)	120 (29.3)	283 (69.0)	127 (31.0)	0.24^***^	0.14–0.34
13. When I eat a portion of dairy products, they are always natural (milk, yogurt without sugar, fresh cheese) or minimally processed	294 (72.1)	114 (27.9)	261 (64.0)	147 (36.0)	0.21^***^	0.11–0.31
14. When I have breakfast, I eat pastries, cookies, juices, smoothies or processed products	240 (58.5)	170 (41.5)	230 (56.1)	180 (43.9)	0.47^***^	0.39–0.56
15. When I eat breakfast, I eat unprocessed or minimally processed foods (fruit, nuts, eggs, or whole wheat bread)	131 (32.0)	279 (68.0)	128 (31.2)	282 (68.8)	0.37^***^	0.27–0.47
16. I eat industrial pastries (sweets, cookies, snacks, or chocolate) and sweets (crisps, worms, candies, or jellies) several times a week	265 (64.6)	145 (35.4)	293 (71.5)	117 (28.5)	0.47^***^	0.67–0.85
**KIDMED 2.0 index score**				
Poor ( ≤ 3)	123 (30.0)		282 (68.8)		0.17^***^	0.11–0.23
Average (4–7)	218 (53.2)		115 (28.0)			
Good (≥8)	69 (16.8)		13 (3.2)			

Significant results:

* p < 0.05,

** p < 0.01,

*** p < 0.001.

## Discussion

This research aims to (i) update the 2019 KIDMED questionnaire; and (ii) test its psychometric properties in terms of reliability and validity in a group of Spanish children and adolescents. Concerning the first aim, and following the modifications of the current version of KIDMED 2.0, the changes in each item are shown, justifying them from a scientific point of view.

In this sense, an update was proposed for Items 1 and 2 which previously referred to: “Eats a fruit every day” and “Eats a second fruit every day,” respectively. The World Health Organization (48) recommends eating at least 400 g of fruit and vegetables per day, which is equivalent to approximately 5 servings per day. Previous research has shown that fruit and vegetable consumption is associated with a lower risk of non-communicable diseases (49–51). In addition, meeting fruit and vegetable consumption recommendations reduces the likelihood of becoming overweight and obese (49, 51). Increased fruit and vegetable consumption could help weight loss in children and adolescents (52), the target populations of KIDMED 2.0. Therefore, rephrasing Items 1 and 2 was proposed as follows: “Eats two or more servings of fruit a day” and “Takes one or more servings of vegetables and/or raw or cooked vegetables per day,” respectively.

Item 4 of the Altavilla and Caballero-Perez (5) research questionnaire stated “Has fresh or cooked vegetables more than once per day.” Given that it is recommended to consume a large amount of vegetables and fruits daily to achieve health benefits, we considered rephrasing Item 6 as follows: “More than half of the food he/she eats is of plant origin (fruits, vegetables, legumes, nuts, potatoes, whole grains).” Previous research has suggested that fruit and vegetable consumption is the fundamental basis of the Mediterranean diet (1, 53).

Items 5 and 7, also of the Altavilla and Caballero-Perez (5) questionnaire, indicated: “Consumes fish regularly (at least 2–3 times per week)” and “Likes pulses and eats them more than once per week,” respectively. In the case of pulses, they have been shown to have excellent nutritional value and are an important source of fiber, phytosterols, and various minerals (54). Fish, especially blue fish, contains abundant omega-3 polyunsaturated fatty acids such as docosahexaenoic acid and eicosapentaenoic acid, which can improve cognitive function and prevent cardiovascular disease (55). As red meat consumption has been associated with an increased risk of colourectal cancer, it is recommended to preferably consume lean meat (poultry and rabbit) (54, 56). Therefore, it was proposed to rephrase Items 3, 5, and 7 as follows: “Eats one portion at lunch and another at dinner of legumes, meat (chicken, turkey, or rabbit or other lean meats), fish and/or eggs a day,” “When he/she eats lean meat, eggs and/or fish, they are usually fresh and minimally processed,” and “Eats three or more servings of legumes (chickpeas, white beans, lentils, peas) a week,” respectively.

Item 6 of the questionnaire of Altavilla and Caballero-Perez (5), which indicated “Goes to a fast-food (hamburger) restaurant more than once per week” was also modified. Ultra-processed foods are characterized by a high content of added sugars, salt, dietary energy density, along with saturated and trans fats, and low amounts of fiber, protein, micronutrients, and phytochemicals (57, 58). The systematic review and meta-analyses conducted by Askari et al. (58) showed a positive association between the consumption of ultra-processed foods and excess body weight. The composition of these foods, also known as “ready-to-eat,” or “ready-to-heat,” is very similar to fast foods, as they are created from industrial formulations manufactured from substances derived from foods and additives, with minimal whole food (58). Therefore, it was proposed to rephrase Item 6 as follows: “Eats pre-cooked food or fast foods, such as pizzas and hamburgers, one or more times a week.”

In 2010, the UNESCO definition of the Mediterranean diet includes cooking skills as a fundamental aspect of the Mediterranean pattern. The Mediterranean diet effect on health may differ between Mediterranean and non-Mediterranean countries (59), possibly because the normal concept of transferability is only about food consumption and less about other aspects, such as food consumption during the day, and culinary customs (60). Culinary habits and skills are important aspects of lifestyle and may be related to better food consumption because when people cook their meals, they are more careful about what they consume (59, 60). This approach shows that what you eat is important but how you eat is more so (i.e., how you prepare the food). However, the measurement in previous versions of KIDMED has only taken into account the mode of preparation for vegetables. Therefore, for the KIDMED 2.0 version, Item 8 was included to assess the mode of food preparation, which reads: “At home, it is usually cooked in the oven, grilled (a frying pan with little oil) or cooked (not using a fryer).”

Concerning Item 9, Altavilla and Caballero-Perez (5) indicated “Consumes whole-grain pasta or whole-grain rice almost every day (5 or more times per week).” Whole-grain cereals are richer in vitamins, minerals, antioxidants, and dietary fiber than refined cereals, which may have a protective effect against cancers of the digestive system (61, 62). As Item 9 of Altavilla and Caballero-Perez (5) already included the term “whole-grain” in the consumption of whole-grain pasta or whole-grain rice, no significant changes were proposed for this item. Therefore, Item 9 reads as follows: “When I eat cereals (pasta, rice, quinoa, couscous), I always eat whole grains.” It would be given a value of +1 because we consider the consumption of whole-grain cereals as a healthy eating habit within the framework of the Mediterranean diet.

As for Item 10 of the Altavilla and Caballero-Perez (5) version, it says: “Consumes nuts regularly (at least 2–3 times per week.” Regular consumption of nuts (especially almonds and walnuts) has been associated with a lower risk of coronary heart disease (54). The cardioprotective effects of nuts may be related to their relatively high proportion of unsaturated fatty acids such as monounsaturated fatty acids and linoleic acid (63). However, if nuts are fried, they may contain high levels of salt and oil, which may reduce their protective effect. Therefore, we decided to include the term “natural” to clarify the mode of consumption of nuts, so that Item 10 was worded: “Has a serving of natural or roasted nuts without salt at least 3 times a week.”

Regarding Item 13, the Altavilla and Caballero-Perez (5) KIDMED said: “Has a dairy product for breakfast (yogurt, milk, etc.).” Consumption of dairy products may reduce the risk of cardiovascular disease (64, 65). To meet dietary recommendations for dairy foods and calcium intake, it is recommended to consume 2.5 to 4 servings of dairy products per day (48). As the benefit of consuming these foods is independent of the time of day when they are consumed, it was proposed to remove the word “breakfast” from Altavilla and Caballero-Perez (5) questionnaire, so that the wording of the item would read as follows: “When he/she eats a portion of dairy products, they are always natural (e.g., milk, yogurt without sugar, fresh cheese) or minimally processed.”

Item 11 of the Altavilla and Caballero-Perez (5) version indicates “Uses olive oil at home.” Strong evidence has demonstrated the protective effect of virgin olive oil (53). Moreover, the Mediterranean diet is characterized by the consumption of virgin olive oil as one of the main sources of fat (1, 8), which justifies its inclusion in the KIDMED 2.0. However, the wording of the item may be confusing for the respondent, so it was modified to make it easier to understand for children and adolescents, now reading: “At home, virgin olive oil (dark green) is used instead of sunflower oil (yellow).” It would be given a value of +1, as it is considered a fundamental food in the Mediterranean diet.

Regarding breakfast, the Altavilla and Caballero-Perez (5) questionnaire indicated: “Skips breakfast.” Eating breakfast is considered an essential feature of a Mediterranean dietary pattern. It has been shown that children and adolescents who ate breakfast regularly were more likely to adhere to the Mediterranean diet than those who did not eat breakfast (66). However, there is growing evidence to question this position, arguing that the beneficial effects of breakfast depend on the type of food we eat (67). Studies on the association of breakfast intake are rather limited (67), so, in line with this approach, two different questions referring to breakfast were proposed, one referring to healthy foods and the other to unhealthy foods. Thus, the wording of Items 14 and 15 were proposed as follows: “When he/she has breakfast, he/she eats pastries, cookies, juices, smoothies or processed products” and “When he/she eats breakfast, he/she eats unprocessed or minimally processed foods (e.g., fruit, nuts, eggs or whole wheat bread),” respectively.

Item 16 of the Altavilla and Caballero-Perez (5) version reads: “Eats sweets and candy several times every day.” Sweets and candy are an important source of salt, trans fats, and sugar, which have been associated with weight gain (54). Furthermore, the systematic review conducted by Iaccarino-Idelson et al. (9) defined salty snacks, sweets, candies, soft drinks, juices, and/or shakes as “anti-Mediterranean” food. To facilitate the understanding of this item, some examples were proposed. Thus, the wording of Items 12 and 16 is: “Drinks commercial soft drinks, juices and/or shakes one or more times during the week” and “Eats industrial pastries (sweets, cookies, snacks or chocolate) and sweets (crisps, worms, candies or jellies) several times a week,” respectively. Finally, content validity was accepted by reaching a full agreement after completing all phases of the process.

Regarding KIDMED's reliability, Cohen's kappa coefficients revealed adequate reliability (47), which confirms the good consistency of KIDMED 2.0. These values are similar to those reported by Carrillo and Ramírez-Vélez (32), and Rei et al. (21), which showed good agreement in almost every item and in the KIDMED index score. Also, results showed similar values between males and females, as well as between children and adolescents. Thus, the KIDMED 2.0 is presented as a reliable questionnaire to be used regardless of sex and age in the early stages.

Concerning construct validity, our adaptation of the KIDMED showed acceptable validity (47). These values are similar to those of Rei et al. (21), who reported moderate validity of the KIDMED questionnaire in Portuguese adolescents. In the present study, only Items 3, 8, and 11 showed poor agreement. One of the main reasons for the low values in Items 8 and 11 was that the participants did not indicate the type of cooking and the type of oil used by their families in the 7-day DR. The results tested in Item 3 may also be due to the general descriptions of the indicated dishes, especially typical foods such as purees or different stews. For instance, purees can be made only of vegetables or also of protein products such as chicken or beef. Therefore, more studies with different samples and/or contexts are needed to check the construct validity of these items.

### Limitations, future directions, and strengths

The present investigation has several limitations that should be taken into account when interpreting the results. First, according to the poor values of construct validity for Items 3, 8, and 11, the 7-day DR selected as the gold standard measurement should be improved, obtaining a more detailed record of meals. Therefore, in future studies, it would be interesting to conduct a joint assessment of the 7-day DR of parents and children to gain more knowledge about consumption that would improve and provide higher quality to the scientific instrument. Another limitation of this project is that we only included children and adolescents from one region of Spain. Future studies should examine the psychometric properties of the KIDMED 2.0 in different regions or countries where the Mediterranean diet is practiced and rooted.

Despite these limitations, the present study has some strengths. Firstly, our study carries out an in-depth review of each item according to Mediterranean dietary patterns during the adaptation and validation process of the KIDMED 2.0, supported and supervised by a team of nutritionists who are experts in the Mediterranean diet. Secondly, our study analyses the psychometric properties of the KIDMED in Spanish adolescents using a mixed methodology (i.e., a quantitative and qualitative methodology were used conjointly). Only one previous study has analyzed the psychometric properties in Portuguese adolescents (21). Finally, the reliability and validity of the KIDMED 2.0 according to age and sex were also tested.

## Conclusions

This study provides a new instrument to assess the adherence of children and adolescents to the Mediterranean diet, carrying out a thorough revision of the items to adapt the instrument to the real Mediterranean diet. Moreover, this adaptation has undergone a rigorous analysis of its psychometric properties, obtaining moderate validity and reliability. Therefore, the KIDMED 2.0 should be further refined. Finally, researchers and teachers from different educational centers interested in this topic will be able to evaluate adherence to the Mediterranean diet with greater rigor.

## Data availability statement

The raw data supporting the conclusions of this article will be made available by the authors, without undue reservation.

## Ethics statement

The studies involving human participants were reviewed and approved by the Bioethics Committee of the University of the Extremadura 239/2019. Written informed consent to participate in this study was provided by the participants' legal guardian/next of kin.

## Author contributions

FL and PS-M performed the study, acquired and interpreted the data, and drafted the first version of the manuscript. DL-G and CS interpreted the data and improved the manuscript. ML-G and MT-S designed the study, interpreted the data, had overall responsibility for the study, and improved the manuscript. All authors approved the final manuscript.

## Funding

This work was supported by Junta de Extremadura (Ministry of Economy, Science and Digital Agenda) with the contribution of the European Union through the European Regional Development Fund Infrastructure, and Government of Spain (Ministry of Education, Culture and Sports, IJC2019-040788-I). ML-G is supported by the Government of Spain (Ministry of Education, Culture and Sports, FPU17/03489). MT-S is supported by the Junta 432 de Extremadura (PD18015) and European Social Fund (FSE).

## Conflict of interest

Author DL-G was employed by the company Cliniber–Diet Therapy and Nutritional Education (Cliniber–Nutrición, Dietoterapia y Coaching Nutricional). Author CS was employed by the company Nut&Cook. The remaining authors declare that the research was conducted in the absence of any commercial or financial relationships that could be construed as a potential conflict of interest.

## Publisher's note

All claims expressed in this article are solely those of the authors and do not necessarily represent those of their affiliated organizations, or those of the publisher, the editors and the reviewers. Any product that may be evaluated in this article, or claim that may be made by its manufacturer, is not guaranteed or endorsed by the publisher.

## References

[1] Cabrera SG Herrera Fernández N Rodríguez Hernández C Nissensohn M Román-Viñas B Serra-Majem L. KIDMED test; prevalence of low adherence to the Mediterranean Diet in children and young; a systematic review. Nutr Hosp. (2015) 32:2390–9. 10.3305/nh.2015.32.6.9828 26667685

[2] Serra-Majem L Ortiz-Andrellucchi A. The Mediterranean diet as an example of food and nutrition sustainability: a multidisciplinary approach. Nutrición Hospitalaria. (2018) 35:96–101. 10.20960/nh.2133 30070130

[3] Donini LM Serra-Majem L Bulló M Gil Á Salas-Salvadó J. The Mediterranean diet: culture, health and science. Br J Nutr. (2015) 113:S1–3. 10.1017/S0007114515001087 26148911

[4] Altavilla C Comeche JM Comino Comino I Caballero Pérez P. Spanish update of the Kidmed questionnaire, a mediterranean diet quality index in children and adolescents. Rev Esp Salud Publica. (2020) 94:e202006057. 32555141 PMC11582840

[5] Altavilla C Caballero-Pérez P. An update of the KIDMED questionnaire, a Mediterranean Diet Quality Index in children and adolescents. Public Health Nutr. (2019) 22:2543–7. 10.1017/S1368980019001058 31146796 PMC10260407

[6] Guthold R Stevens GA Riley LM Bull FC. Global trends in insufficient physical activity among adolescents: a pooled analysis of 298 population-based surveys with 1·6 million participants. Lancet Child Adolesc Health. (2020) 4:23–35. 10.1016/S2352-4642(19)30323-2 31761562 PMC6919336

[7] Garrido-Miguel M Cavero-Redondo I Álvarez-Bueno C Rodríguez-Artalejo F Moreno LA Ruiz JR. Prevalence and trends of overweight and obesity in European children from 1999 to 2016. JAMA Pediatr. (2019) 173:e192430. 10.1001/jamapediatrics.2019.2430 31381031 PMC6686782

[8] Serra-Majem L Ribas L Ngo J Ortega RM García A Pérez-Rodrigo C. Food, youth and the Mediterranean diet in Spain. Development of KIDMED, Mediterranean Diet Quality Index in children and adolescents. Public Health Nutr. (2004) 7:931–5. 10.1079/PHN2004556 15482620

[9] Iaccarino-Idelson P Scalfi L Valerio G. Adherence to the Mediterranean Diet in children and adolescents: a systematic review. Nutr Metabol Cardiovasc Dis. (2017) 27:283–99. 10.1016/j.numecd.2017.01.002 28254269

[10] Aparicio-Ugarriza R Cuenca-García M Gonzalez-Gross M Julián C Bel-Serrat S Moreno LA. Relative validation of the adapted Mediterranean Diet Score for Adolescents by comparison with nutritional biomarkers and nutrient and food intakes: the Healthy Lifestyle in Europe by Nutrition in Adolescence (HELENA) study. Public Health Nutr. (2019) 22:2381–97. 10.1017/S1368980019001022 31204628 PMC10260553

[11] Novak D Štefan L Prosoli R Emeljanovas A Mieziene B Milanović I. Mediterranean Diet and its correlates among adolescents in non-Mediterranean European countries: a population-based study. Nutrients. (2017) 9:177. 10.3390/nu9020177 28241432 PMC5331608

[12] Kontogianni MD Farmaki AE Vidra N Sofrona S Magkanari F Yannakoulia M. Associations between lifestyle patterns and body mass index in a sample of Greek Children and adolescents. J Am Diet Assoc. (2010) 110:215–21. 10.1016/j.jada.2009.10.035 20102848

[13] Vassiloudis I Yiannakouris N Panagiotakos DB Apostolopoulos K Costarelli V. Academic performance in relation to adherence to the Mediterranean diet and energy balance behaviors in Greek primary schoolchildren. J Nutr Educ Behav. (2014) 46:164–70. 10.1016/j.jneb.2013.11.001 24433816

[14] Tambalis KD Panagiotakos DB Psarra G Sidossis LS. Current data in Greek children indicate decreasing trends of obesity in the transition from childhood to adolescence; results from the National Action for Children's Health (EYZHN) program. J Prev Med Hyg. (2018) 59:E36. 10.15167/2421-4248/jpmh2018.59.1.797 29938238 PMC6009074

[15] Peng W Goldsmith R Berry EM. Demographic and lifestyle factors associated with adherence to the Mediterranean diet in relation to overweight/obesity among Israeli adolescents: findings from the Mabat Israeli national youth health and nutrition survey. Public Health Nutr. (2017) 20:883–92. 10.1017/S1368980016002779 27829478 PMC10261575

[16] Grassi T Bagordo F Panico A Giorgi M de Idolo A Serio F. Adherence to Mediterranean diet of children living in small Southern Italian villages. Int J Food Sci Nutr. (2019) 71:490–9. 10.1080/09637486.2019.1679725 31631719

[17] Mistretta A Marventano S Antoci M Cagnetti A Giogianni G Nolfo F. Mediterranean diet adherence and body composition among Southern Italian adolescents. Obes Res Clin Pract. (2017) 11:215–26. 10.1016/j.orcp.2016.05.007 27269367

[18] Santomauro F Lorini C Tanini T Indiani L Lastrucci V Comodo N. Adherence to Mediterranean diet in a sample of Tuscan adolescents. Nutrition. (2014) 30:1379–83. 10.1016/j.nut.2014.04.008 25280416

[19] Mounayar R Jreij R Hachem J Abboud F Tueni M. Breakfast intake and factors associated with adherence to the Mediterranean diet among Lebanese high school adolescents. J Nutr Metabol. (2019) 2019:2714286. 10.1155/2019/2714286 31275644 PMC6589235

[20] Leal FMR Oliveira BMPM Pereira SSR. Relationship between cooking habits and skills and Mediterranean diet in a sample of Portuguese adolescents. Perspect Public Health. (2011) 131:283–7. 10.1177/1757913911419909

[21] Rei M Severo M Rodrigues S. Reproducibility and validity of the Mediterranean Diet Quality Index (KIDMED Index) in a sample of Portuguese adolescents. Br J Nutr. (2021) 126:1737–48. 10.1017/S0007114521000532 33583437

[22] Rito AI Dinis A Rascôa C Maia A Mendes S Stein-Novais C. Mediterranean Diet Index (KIDMED) adherence, socioeconomic determinants, and nutritional status of Portuguese children: the eat Mediterranean program. Port J Public Health. (2018) 36:141–9. 10.1159/000495803

[23] Arcila-Agudelo AM Ferrer-Svoboda C Torres-Fernàndez T Farran-Codina A. Determinants of adherence to healthy eating patterns in a population of children and adolescents: evidence on the Mediterranean diet in the city of Mataró (Catalonia, Spain). Nutrients. (2019) 11:854. 10.3390/nu11040854 30991741 PMC6520885

[24] Galan-Lopez P Sánchez-Oliver AJ Ries F González-Jurado JA. Mediterranean diet, physical fitness and body composition in sevillian adolescents: a healthy lifestyle. Nutrients. (2019) 11:2009. 10.3390/nu11092009 31454923 PMC6769614

[25] Morales-Suárez-Varela M Clemente-Bosch E Peraita-Costa I Llopis-Morales A Martínez I Llopis-González A. Maternal physical activity during pregnancy and the effect on the mother and newborn: a systematic review. J Phys Activity Health. (2021) 18:130–47. 10.1123/jpah.2019-0348 33361475

[26] Tapia-Serrano MA Vaquero-Solís M López-Gajardo MA Sánchez-Miguel PA. Adherencia a la dieta mediterránea e importancia de la actividad física y el tiempo de pantalla en los adolescentes extremeños de enseñanza secundaria. Nutr Hosp. (2021) 38:236–44. 10.20960/nh.03372 33319582

[27] Sahingoz SA Sanlier N. Compliance with Mediterranean Diet Quality Index (KIDMED) and nutrition knowledge levels in adolescents. A case study from Turkey. Appetite. (2011) 57:272–7. 10.1016/j.appet.2011.05.307 21624407

[28] Cakir M Akbulut UE Okten A. Association between adherence to the Mediterranean diet and presence of nonalcoholic fatty liver disease in children. Child Obes. (2016) 12:279–85. 10.1089/chi.2015.0197 26871614

[29] Apaydin Kaya Ç Temiz G. The Turkish version of the Mediterranean diet quality index (KIDMED). TJFMPC. (2021) 15:341–7. 10.21763/tjfmpc.836560

[30] Simon MISS Forte GC Marostica PJC. Translation and cultural adaptation of the Mediterranean diet quality index in children and adolescents. Rev Paul Pediat. (2020) 38:e2018242. 10.1590/1984-0462/2020/38/2018242 31939514 PMC6958546

[31] Garcia-Hermoso A Vegas-Heredia ED Fernández-Vergara O Ceballos-Ceballos R Andrade-Schnettler R Arellano-Ruiz P. Independent and combined effects of handgrip strength and adherence to a Mediterranean diet on blood pressure in Chilean children. Nutrition. (2019) 60:170–4. 10.1016/j.nut.2018.08.019 30611079

[32] Carrillo HA Ramírez-Vélez R. Adherencia a la dieta mediterránea en una población escolar colombiana: evaluación de las propiedades psicométricas del cuestionario KIDMED. Nutricion hospitalaria. (2020) 37:73–9. 10.20960/nh.02760 31746624

[33] Lazarou C Panagiotakos DB Matalas AL. Physical activity mediates the protective effect of the Mediterranean diet on children's obesity status: the CYKIDS study. Nutrition. (2010) 26:61–7. 10.1016/j.nut.2009.05.014 19632093

[34] Galan-Lopez P Sanchez-Oliver AJ Pihu M Gísladóttír T Domínguez R Ries F. Association between adherence to the mediterranean diet and physical fitness with body composition parameters in 1717 European adolescents: the adoleshealth study. Nutrients. (2019) 12:77. 10.3390/nu12010077 31892139 PMC7019378

[35] Galan-Lopez P Domínguez R Pihu M Gísladóttir T Sánchez-Oliver AJ Ries F. Evaluation of physical fitness, body composition, and adherence to Mediterranean diet in adolescents from Estonia: the AdolesHealth study. Int J Environ Res Public Health. (2019) 16:4479. 10.3390/ijerph16224479 31739416 PMC6888343

[36] Eloranta AM Schwab U Venäläinen T Kiiskinen S Lakka HM Laaksonen DE. Dietary quality indices in relation to cardiometabolic risk among Finnish children aged 6–8 years – The PANIC study. Nutr Metabol Cardiovasc Dis. (2016) 26:833–41. 10.1016/j.numecd.2016.05.005 27397511

[37] Kim RJ Lopez R Snair M Tang A. Mediterranean diet adherence and metabolic syndrome in US adolescents. Int J Food Sci Nutr. (2020) 72:537–47. 10.1080/09637486.2020.1840533 33115263

[38] Jennings A Welch A van Sluijs EMF Griffin SJ Cassidy A. Diet quality is independently associated with weight status in children aged 9–10 years. J Nutr. (2011) 141:453–9. 10.3945/jn.110.131441 21270356

[39] Martin-Calvo N Chavarro JE Falbe J Hu FB Field AE. Adherence to the Mediterranean dietary pattern and BMI change among US adolescents. Int J Obes. (2016) 40:1103–8. 10.1038/ijo.2016.59 27102053 PMC4935550

[40] Gehlbach H Brinkworth ME. Measure twice, cut down error: a process for enhancing the validity of survey scales. Rev Gen Psychol. (2011) 15:380–7. 10.1037/a0025704

[41] Dunn JGH Bouffard M Rogers WT. Assessing item content-relevance in sport psychology scale-construction research: Issues and recommendations. Meas Phys Educ Exerc Sci. (1999) 3:15–36. 10.1207/s15327841mpee0301_2

[42] Patton MQ. Two decades of developments in qualitative inquiry: A personal, experiential perspective. Qual Soc Work. (2002) 1:261–83. 10.1177/1473325002001003636

[43] Sparkes AC Smith B. Qualitative research methods in sport, exercise and health: From process to product. Routledge (2014).

[44] Suchindran C. Sample size sample size. Sampling and Choosing Cases in Qualitative Research: A Realist Approach. (2014). p. 2–6.

[45] Ortega RM Pérez-Rodrigo C López-Sobaler AM. Dietary assessment methods: dietary records. Nutr Hosp. (2015) 31 (Suppl 3):38–45. 10.3305/nh.2015.31.sup3.8749 25719769

[46] Štefan L Prosoli R Juranko D Cule M Milinović I Novak D. The reliability of the mediterranean diet quality index (KIDMED) questionnaire. Nutrients. (2017) 9:419. 10.3390/nu9040419 28441742 PMC5409758

[47] Landis JR Koch GG. The measurement of observer agreement for categorical data. Biometrics. (1977) 33:159. 10.2307/2529310 843571

[48] World Health Organization. Healthy Diet. (2020). Available online at: https://www.who.int/news-room/fact-sheets/detail/healthy-diet (accessed February 15, 2022).

[49] Vereecken C Pedersen TP Ojala K Krølner R Dzielska A Ahluwalia N. Fruit and vegetable consumption trends among adolescents from 2002 to 2010 in 33 countries. Eur J Public Health. (2015) 25:16–9. 10.1093/eurpub/ckv012 25805780

[50] Wang P Fang J Gao Z Zhang C Xie S. Higher intake of fruits, vegetables or their fiber reduces the risk of type 2 diabetes: a meta-analysis. J Diabetes Investig. (2016) 7:56–69. 10.1111/jdi.12376 26816602 PMC4718092

[51] Darfour-Oduro SA Buchner DM Andrade JE Grigsby-Toussaint DS. A comparative study of fruit and vegetable consumption and physical activity among adolescents in 49 Low-and-Middle-Income Countries OPEN. Sci Rep. (2018) 8:1623. 10.1038/s41598-018-19956-0 29374197 PMC5785955

[52] Wojcicki JM Heyman MB. Reducing childhood obesity by eliminating 100% fruit juice. Am J Public Health. (2012) 102:1630–3. 10.2105/AJPH.2012.300719 22813423 PMC3482038

[53] Martinez-Gonzalez MA Martin-Calvo N. Mediterranean diet and life expectancy; beyond olive oil, fruits, and vegetables. Curr Opin Clin Nutr Metab Care. (2016) 19:401–7. 10.1097/MCO.0000000000000316 27552476 PMC5902736

[54] Hoffman R Gerber M. Evaluating and adapting the Mediterranean diet for non-Mediterranean populations: a critical appraisal. Nutr Rev. (2013) 71:573–84. 10.1111/nure.12040 24032362

[55] Martínez-Lapiscina EH Clavero P Estruch R Martínez-Lapiscina EH Toledo E Salas-Salvadó J. Mediterranean diet improves cognition: the PREDIMED-NAVARRA randomised trial Cardiovascular Risk and aging View project. J Neurol. (2013). 10.1136/jnnp-2012-304792 23670794

[56] Formisano E Pasta A Cremonini AL di Lorenzo I Sukkar SG Pisciotta L. Effects of a Mediterranean diet, dairy, and meat products on different phenotypes of dyslipidemia: a preliminary retrospective analysis. Nutrients. (2021) 13:1161. 10.3390/nu13041161 33915861 PMC8065939

[57] Bielemann RM Motta JVS Minten GC Horta BL Gigante DP. Consumption of ultra-processed foods and their impact on the diet of young adults. Rev Saúde Pública. (2015) 49:1–10. 10.1590/S0034-8910.2015049005572 26018785 PMC4560335

[58] Askari M Heshmati J Shahinfar H Tripathi N Daneshzad E. Ultra-processed food and the risk of overweight and obesity: a systematic review and meta-analysis of observational studies. Int J Obes. (2020) 44:2080–91. 10.1038/s41366-020-00650-z 32796919

[59] Mocciaro G Ziauddeen N Godos J Marranzano M Chan MY Ray S. Does a Mediterranean-type dietary pattern exert a cardio-protective effect outside the Mediterranean region? A review of current evidence. Int J Food Sci Nutr. (2018) 69:524–35. 10.1080/09637486.2017.1391752 29063806

[60] Real H Queiroz J Graça P. Mediterranean food pattern vs. Mediterranean diet: a necessary approach? Int J Food Sci Nutr. (2020) 71:1–12. 10.1080/09637486.2019.1617838 31122086

[61] Marventano S Vetrani C Vitale M Godos J Riccardi G Grosso G. Whole grain intake and glycaemic control in healthy subjects: a systematic review and meta-analysis of randomized controlled trials. Nutrients. (2017) 9:769. 10.3390/nu9070769 28753929 PMC5537883

[62] Quick V Wall M Larson N Haines J Neumark-Sztainer D. Personal, behavioral and socio-environmental predictors of overweight incidence in young adults: 10-yr longitudinal findings. Int J Behav Nutr Phys Act. (2013) 10:37. 10.1186/1479-5868-10-37 23531253 PMC3623851

[63] Ros E Mataix J. Fatty acid composition of nuts – implications for cardiovascular health. Br J Nutr. (2006) 96:S29–35. 10.1017/BJN20061861 17125530

[64] Guo J Astrup A Lovegrove JA Gijsbers L Givens DI Soedamah-Muthu SS. Milk and dairy consumption and risk of cardiovascular diseases and all-cause mortality: dose–response meta-analysis of prospective cohort studies. Eur J Epidemiol. (2017) 32:269–87. 10.1007/s10654-017-0243-1 28374228 PMC5437143

[65] Drouin-Chartier JP Brassard D Tessier-Grenier M Côté JA Labonté MÈ Desroches S. Systematic review of the association between dairy product consumption and risk of cardiovascular-related clinical outcomes. Adv Nutr Int Rev J. (2016) 7:1026–40. 10.3945/an.115.011403 28140321 PMC5105032

[66] Hoyland A Dye L Lawton CL. A systematic review of the effect of breakfast on the cognitive performance of children and adolescents. Nutr Res Rev. (2009) 22:220–43. 10.1017/S0954422409990175 19930787

[67] Lazarou C Matalas AL. Breakfast intake is associated with nutritional status, Mediterranean diet adherence, serum iron and fasting glucose: the CYFamilies study. Public Health Nutr. (2015) 18:1308–16. 10.1017/S1368980014001967 25287356 PMC10271461

