# Supplementary material for: KIDMED 2.0, An update of the KIDMED questionnaire: Evaluation of the psychometric properties in youth
Source: Front Nutr. 2022 Nov 8;9:945721. doi: 10.3389/fnut.2022.945721 (PMC9679638; doi:10.3389/fnut.2022.945721)
Supplement: Supplementary file 1 [file Data_Sheet_1.docx]

**KIDMED 2.0; An Update of the KIDMED Questionnaire: Evaluation of the Psychometric Properties in Children and Adolescents**

Miguel A. López-Gajardo, Francisco M. Leo, Pedro Antonio Sánchez-Miguel, Dori López-Gajardo, Candelaria Soulas, and Miguel A. Tapia-Serrano

*Online Supplementary Files*

| Score | Items |
| --- | --- |
| +1 | 1. I eat a fruit every day |
| +1 | 2. I eat a second fruit every day |
| +1 | 3. I eat fresh (salad) or cooked vegetables regularly once a day |
| +1 | 4. I eat fresh or cooked vegetables more than once a day |
| +1 | 5. I eat fish regularly (at least 2-3 times a week) |
| -1 | 6. I eat precooked food or fast-food (fast food) such as pizzas and hamburgers one or more times a week |
| +1 | 7. I eat one or more servings of legumes a week |
| +1 | 8. I eat whole grain pasta or brown rice more than once time a week |
| +1 | 9. I eat whole grains or whole grain derivatives (whole grain bread, etc.) |
| +1 | 10. I eat nuts regularly (at least 2-3 times a week) |
| +1 | 11. At home, olive oil is used |
| -1 | 12. I do not eat breakfast |
| +1 | 13. Usually I drink a natural dairy (milk, natural yoghurt without sugar, etc.) |
| -1 | 14. I eat sweets and treats several times a day |
| +1 | 15. I eat two yoghurts and/or 40 g of cheese every day |
| -1 | 16. I breakfast industrial pastries, cookies or cupcakes |

# Supplementary Table 1. Initial Version of the KIDMED 2.0 by the Authors

*Note.* alue of the KIDMED 2.0 score: ≤ 3, poor quality diet; 4–7, average quality diet; ≥8, good Mediterranean diet.

| Score | Items |
| --- | --- |
| +1 | 1. I eat **two or more** **servings of** fruit **a** day |
| +1 | 2. I eat **one or more** servings of vegetables **and/or raw or cooked vegetables per day** |
| +1 | 3. **I eat one portion at lunch and another at dinner of legumes, meat (chicken, turkey or rabbit), fish and/or eggs a day** |
| +1 | 4. **More than half of the food I eat is of plant origin (fruits, vegetables, legumes, nuts, potatoes, whole grains)** |
| +1 | 5. **When I eat lean meat, eggs and/or fish, they are usually fresh and minimally processed** |
| -1 | 6. I eat precooked food or fast-food such as pizzas and hamburgers one or more times a week |
| +1 | 7. I eat **three** or more servings of legumes **(chickpeas, white beans, lentils)** a week |
| +1 | 8. **At home, food is usually cooked in the oven, grilled or boiled (do not use a fryer)** |
| +1 | 9. **When I eat cereals (pasta, rice, quinoa, couscous), I always eat** **whole** grains |
| +1 | 10. **I eat a serving of natural or roasted** nuts **without salt at least 3 times a week** |
| +1 | 11. At home, **virgin** olive oil is used **instead of sunflower oil** |
| -1 | 12. I **usually drink (2 times or more) soft drinks, juices and/or commercial shakes during the week** |
| +1 | 13. **When I eat a portion of** dairy **products, they are always** natural (milk, yoghurt without sugar, etc.) **or minimally processed** |
| -1 | 14. **When I have breakfast, I eat pastries, cookies, juices, smoothies or processed products** |
| +1 | 15. When I eat breakfast, I eat unprocessed or minimally processed foods (fruit, nuts, eggs, whole wheat bread, etc.) |
| -1 | 16. I eat industrial pastries **(sweets, cookies, snacks or chocolate) and sweets (crisps, worms, candies or jellies) several times a week** |

**Supplementary Table 2.** *Second Version of the KIDMED 2.0 by the Authors*

*Note.* Value of the KIDMED 2.0 score: ≤ 3, poor quality diet; 4–7, average quality diet; ≥8, good Mediterranean diet. The changes made concerning the previous version of KIDMED 2.0 are presented in bold.

**Supplemental Table 3.** *Initial Version of the KIDMED 2.0 by the External Panel of Expert Nutritionists*

| Score | Items |
| --- | --- |
| +1 | 1. I eat two or more servings of fruit a day |
| +1 | 2. I eat one or more servings of vegetables and/or raw or cooked vegetables per day |
| +1 | 3. I eat one portion at lunch and another at dinner of legumes, meat (chicken, turkey or rabbit, **or other lean meats**), fish and/or eggs a day |
| +1 | 4. More than half of the food I eat is of plant origin (fruits, vegetables, legumes, nuts, potatoes, whole grains) |
| +1 | 5. When I eat lean meat, eggs and/or fish, they are usually fresh and minimally processed |
| -1 | 6. I eat precooked food or fast-food such as pizzas and hamburgers one or more times a week |
| +1 | 7. I eat three or more servings of legumes (chickpeas, white beans, lentils, **peas**) a week |
| +1 | 8. At home, food is usually cooked in the oven, grilled or boiled (do not use a fryer) |
| +1 | 9. When I eat cereals (pasta, rice, quinoa, couscous), I always eat whole grains |
| +1 | 10. I eat a serving of natural or roasted nuts without salt at least 3 times a week |
| +1 | 11. At home, virgin olive oil is used instead of sunflower oil |
| -1 | 12. I drink commercial soft drinks, juices and/or shakes **one or** more times during the week |
| +1 | 13. When I eat a portion of dairy products, they are always natural (milk, yoghurt without sugar, **fresh cheese**) or minimally processed |
| -1 | 14. When I have breakfast, I eat pastries, cookies, juices, smoothies or processed products |
| +1 | 15. When I eat breakfast, I eat unprocessed or minimally processed foods (fruit, nuts, eggs, or whole wheat bread) |
| -1 | 16. I eat industrial pastries (sweets, cookies, snacks or chocolate) and sweets (crisps, worms, candies, or jellies) several times a week |

*Note.* Value of the KIDMED 2.0 score: ≤ 3, poor quality diet; 4–7, average quality diet; ≥8, good Mediterranean diet. The changes made concerning the previous version of KIDMED 2.0 are presented in bold.

| Score | English version [Spanish version] |
| --- | --- |
| +1 | 1. I eat two or more servings of fruit a day [Tomo dos o más raciones de fruta al día] |
| +1 | 2. I eat one or more servings of vegetables and/or raw or cooked vegetables per day [Tomo una o más raciones de verduras y/o hortalizas crudas o cocinadas al día] |
| +1 | 3. I eat one portion at lunch and another at dinner of legumes, meat (chicken, turkey or rabbit, or other lean meats), fish and/or eggs a day [Tomo una ración en comida y otra en cena de legumbres, carne (pollo, pavo o conejo u otras carnes sin grasas), pescado y/o huevo al día] |
| +1 | 4. More than half of the food I eat is of plant origin (fruits, vegetables, legumes, nuts, potatoes, whole grains) [Más de la mitad de los alimentos que consumo son de origen vegetal (frutas, verduras, hortalizas, legumbres, frutos secos, patatas, cereales integrales)] |
| +1 | 5. When I eat lean meat, eggs and/or fish, they are usually fresh and minimally processed [Cuando consumo carne sin grasa, huevos y/o pescados suelen ser frescos y mínimamente procesados] |
| -1 | 6. I eat precooked food or fast-food such as pizzas and hamburgers one or more times a week [Tomo una o más veces a la semana comida precocinada o comida rápida tipo pizzas y hamburguesas] |
| +1 | 7. I eat three or more servings of legumes (chickpeas, white beans, lentils, peas) a week [Tomo una o más raciones de legumbres (garbanzos, judías blancas, lentejas, guisantes) a la semana] |
| +1 | 8. At home, food is usually cooked in the oven, grilled **(a frying pan with little oil)** or boiled (do not using a fryer) [En casa se suele cocinar al horno, a la plancha (sartén con poco aceite) o cocido (no utilizar freidora)] |
| +1 | 9. When I eat cereals (pasta, rice, quinoa, couscous), I always eat whole grains [Cuando tomo cereales (pasta, arroz, quinoa, cuscús) siempre los consumos integrales] |
| +1 | 10. I eat a serving of natural or roasted nuts without salt at least 3 times a week [Tomo una ración de frutos secos naturales o tostados sin sal al menos 3 veces a la semana] |
| +1 | 11. At home, virgin olive oil **(dark green)** is used instead of sunflower oil **(yellow)** [En casa se utiliza el aceite de oliva virgen (verde oscuro) en lugar del aceite de girasol (amarillo)] |
| -1 | 12. I drink commercial soft drinks, juices and/or shakes one or more times during the week [Tomo una o más veces refrescos, zumos y/o batidos comerciales durante la semana] |
| +1 | 13. When I eat a portion of dairy products, they are always natural (milk, yoghurt without sugar, fresh cheese) or minimally processed [Cuando tomo una ración de lácteos son siempre naturales (leche, yogurt sin azúcar, queso fresco) o mínimamente procesados] |
| -1 | 14. When I have breakfast, I eat pastries, cookies, juices, smoothies or processed products [Cuando desayuno tomo bollería, galletas, zumos, batidos o productos procesados] |
| +1 | 15. When I eat breakfast, I eat unprocessed or minimally processed foods (fruit, nuts, eggs, or whole wheat bread) [Cuando desayuno tomo alimentos sin procesar o mínimamente procesados (fruta, frutos secos, huevo o pan integral)] |
| -1 | 16. I eat industrial pastries (sweets, cookies, snacks, or chocolate) and sweets (crisps, worms, candies, or jellies) several times a week [Tomo varias veces a la semana bollería industrial (dulces, galletas, snacks o chocolate) y golosinas (patatas fritas, gusanitos, caramelos o gominolas)] |

**Supplemental Table 4.** *Final Version of the KIDMED 2.0*

*Note.* Value of the KIDMED 2.0 score: ≤ 3, poor quality diet; 4–7, average quality diet; ≥8, good Mediterranean diet. The changes made concerning the previous version of KIDMED 2.0 are presented in bold.

**Supplemental Table 5.** *Test-retest and Reliability Values of the Items of the KIDMED as a function of sex*

| KIDMED 2.0 | Girls (*n* = 234) | | | | Cohen’s  Kappa | 95% CI | Boys (*n* = 185) | | | | Cohen’s  Kappa | 95% CI |  |
| --- | --- | --- | --- | --- | --- | --- | --- | --- | --- | --- | --- | --- | --- |
|  | Baseline test | | Re-Test after 2 weeks | |  |  | Baseline test | | Re-Test after 2 weeks | |  |  |  |
|  | Yes (%) | No (%) | Yes (%) | No (%) |  |  | Yes (%) | No (%) | Yes (%) | No (%) |  |  |  |
| 1. I eat two or more servings of fruit a day | 117 (50.0) | 117 (50.0) | 120 (51.3) | 114 (48.7) | .61^***^ | .51 – .72 | 97 (52.7) | 87 (47.3) | 90 (48.9) | 94 (51.1) | .66^***^ | .55 – .77 |  |
| 2. I eat one or more servings of vegetables and/or raw or cooked vegetables per day | 113 (48.3) | 121 (51.7) | 104 (44.4) | 130 (55.6) | .60^***^ | .50 – .70 | 81 (44.0) | 103 (56.0) | 78 (42.4) | 106 (57.6) | .39^***^ | .25 – .53 |  |
| 3. I eat one portion at lunch and another at dinner of legumes, meat (chicken, turkey or rabbit, or other lean meats), fish and/or eggs a day | 193 (82.5) | 41 (17.5) | 178 (76.1) | 56 (23.9) | .37^***^ | .23 – .51 | 123 (81.5) | 27 (18.5) | 137 (74.5) | 47 (25.5) | .36^***^ | .21 – .52 |  |
| 4. More than half of the food I eat is of plant origin (fruits, vegetables, legumes, nuts, potatoes, whole grains) | 151 (64.5) | 83 (35.5) | 142 (60.7) | 92 (39.3) | .42^***^ | .31 – .55 | 82 (62.5) | 33 (37.5) | 105 (57.1) | 79 (42.9) | .37^***^ | .23 – .51 |  |
| 5. When I eat lean meat, eggs and/or fish, they are usually fresh and minimally processed | 194 (82.9) | 40 (17.1) | 196 (83.8) | 38 (16.2) | .51^***^ | .36 – .66 | 123 (74.5) | 14 (25.5) | 144 (78.3) | 40 (21.7) | .47^***^ | .33 – .62 |  |
| 6. I eat precooked food or fast-food such as pizzas and hamburgers one or more times a week | 122 (52.1) | 112 (47.9) | 129 (55.1) | 105 (44.9) | .54^***^ | .44 – .65 | 103 (56.0) | 81 (44.0) | 112 (60.9) | 72 (39.1) | .50^***^ | .37 – .63 |  |
| 7. I eat three or more servings of legumes (chickpeas, white beans, lentils, peas) a week | 216 (92.3) | 18 (7.7) | 208 (88.9) | 26 (11.1) | .50^***^ | .31 – .69 | 155 (84.2) | 29 (15.8) | 150 (81.5) | 34 (18.5) | .56^***^ | .40 – .72 |  |
| 8. At home, food is usually cooked in the oven, grilled (a frying pan with little oil) or boiled (do not using a fryer) | 206 (88.0) | 28 (12.0) | 211 (90.2) | 23 (9.8) | .32^**^ | .14 – .50 | 138 (86.3) | 25 (13.7) | 153 (83.6) | 30 (16.4) | .25^**^ | .07 – .43 |  |
| 9. When I eat cereals (pasta, rice, quinoa, couscous), I always eat whole grains | 44 (18.8) | 190 (81.2) | 63 (26.9) | 171 (73.1) | .41^***^ | .27– .55 | 52 (28.3) | 132 (71.7) | 58 (31.5) | 126 (68.5) | .56^***^ | .43– .69 |  |
| 10. I eat a serving of natural or roasted nuts without salt at least 3 times a week | 55 (23.5) | 179 (76.5) | 70 (29.9) | 164 (70.1) | .60^***^ | .48 – .72 | 46 (25.1) | 137 (74.9) | 46 (25.1) | 137 (74.9) | .45^***^ | .30 – .60 |  |
| 11. At home, virgin olive oil (dark green) is used instead of sunflower oil (yellow) | 203 (86.8) | 31 (13.2) | 203 (86.8) | 31 (13.2) | .55^***^ | .40 – .71 | 158 (85.9) | 26 (14.1) | 157 (85.3) | 27 (14.7) | .45^***^ | .27 – .63 |  |
| 12. I drink commercial soft drinks, juices and/or shakes one or more times during the week | 154 (65.8) | 80 (34.2) | 157 (67.1) | 77 (32.9) | .70^***^ | .62 – .79 | 139 (75.5) | 45 (24.5) | 119 (64.7) | 65 (35.3) | .46^***^ | .32 – .60 |  |
| 13. When I eat a portion of dairy products, they are always natural (milk, yoghurt without sugar, fresh cheese) or minimally processed | 168 (71.8) | 66 (28.2) | 168 (71.8) | 66 (28.2) | .58^***^ | .46 – .70 | 132 (71.7) | 52 (28.3) | 141 (76.6) | 43 (23.4) | .56^***^ | .42 – .70 |  |
| 14. When I have breakfast, I eat pastries, cookies, juices, smoothies or processed products | 129 (55.1) | 105 (44.9) | 141 (60.3) | 93 (39.7) | .67^***^ | .57 – .77 | 115 (62.5) | 69 (37.5) | 112 (60.9) | 72 (39.1) | .57^***^ | .45 – .70 |  |
| 15. When I eat breakfast, I eat unprocessed or minimally processed foods (fruit, nuts, eggs, or whole wheat bread) | 79 (33.8) | 155 (66.2) | 81 (34.6) | 153 (65.4) | .60^***^ | .50 – .72 | 54 (29.3) | 130 (70.7) | 55 (29.9) | 129 (70.1) | .49^***^ | .35 – .63 |  |
| 16. I eat industrial pastries (sweets, cookies, snacks, or chocolate) and sweets (crisps, worms, candies, or jellies) several times a week | 156 (66.7) | 78 (33.3) | 168 (71.8) | 66 (28.2) | .62^***^ | .51 – .33 | 115 (62.5) | 69 (37.5) | 121 (65.8) | 63 (34.2) | .48^***^ | .35 – .61 |  |
| KIDMED index score | | | | | | | | | | | | | |
| Poor (≤3) | 67 (28.6) | | 78 (32.5) | | .53^***^ | .43 – .63 | 60 (32.4) | | 68 (36.8) | | .41^***^ | .30 – .52 |  |
| Average (4–7) | 128 (55.7) | | 110 (47.0) | |  |  | 95 (51.4) | | 87 (47.0) | |  |  |  |
| Good (≥8) | 39 (16.7) | | 48 (20.5) | |  |  | 30 (16.2) | | 30 (16.2) | |  |  |  |

**Supplemental Table 6.** *KIDMED 2.0 and 7-day Dietary Record Values of the Items as a function of age*

| KIDMED 2.0 | Children (*n* = 207) | | | | Cohen’s  Kappa | 95% CI | Adolescents (*n* = 203) | | | | Cohen’s  Kappa | 95% CI |
| --- | --- | --- | --- | --- | --- | --- | --- | --- | --- | --- | --- | --- |
|  | Baseline test | | 7-day Dietary Record | |  |  | Baseline test | | 7-day Dietary Record | |  |  |
|  | Yes (%) | No (%) | Yes (%) | No (%) |  |  | Yes (%) | No (%) | Yes (%) | No (%) |  |  |
| 1. I eat two or more servings of fruit a day | 121 (58.5) | 86 (41.5) | 104 (50.2) | 103 (49.8) | .27^***^ | .15 – .40 | 91 (44.8) | 112 (55.2) | 104 (51.2) | 99 (48.8) | .46^***^ | .34 – .58 |
| 2. I eat one or more servings of vegetables and/or raw or cooked vegetables per day | 113 (54.6) | 94 (45.4) | 81 (39.1) | 126 (60.9) | .22^**^ | .10 – .35 | 81 (39.9) | 122 (60.1) | 66 (32.5) | 137 (67.5) | .31^***^ | .18 – .44 |
| 3. I eat one portion at lunch and another at dinner of legumes, meat (chicken, turkey or rabbit, or other lean meats), fish and/or eggs a day | 175 (84.5) | 32 (15.5) | 101 (48.8) | 106 (51.2) | .10^*^ | .01 – .20 | 162 (79.8) | 441 (20.2) | 107 (52.7) | 96 (47.3) | .05 | -.06 – .17 |
| 4. More than half of the food I eat is of plant origin (fruits, vegetables, legumes, nuts, potatoes, whole grains) | 138 (66.7) | 69 (33.3) | 88 (42.5) | 119 (57.5) | .09 | -.02 – .22 | 123 (60.6) | 80 (39.4) | 97 (47.8) | 106 (52.2) | .10 | -.03 – .23 |
| 5. When I eat lean meat, eggs and/or fish, they are usually fresh and minimally processed | 165 (79.2) | 87 (20.8) | 143 (69.1) | 64 (30.9) | .12 | .02 – .23 | 161 (79.3) | 42 (20.7) | 135 (66.5) | 68 (33.5) | .14^*^ | .01 – .28 |
| 6. I eat precooked food or fast-food such as pizzas and hamburgers one or more times a week | 98 (47.3) | 109 (52.7) | 172 (83.1) | 35 (16.9) | .19^***^ | .06 – .34 | 122 (60.1) | 81 (39.9) | 153 (75.4) | 50 (24.6) | .15^*^ | .02 – .29 |
| 7. I eat three or more servings of legumes (chickpeas, white beans, lentils, peas) a week | 186 (89.9) | 21 (10.1) | 117 (85.5) | 30 (14.5) | .31^***^ | .13 – .49 | 178 (87.7) | 25 (12.3) | 159 (78.3) | 44 (21.7) | .32^***^ | .17 – .49 |
| 8. At home, food is usually cooked in the oven, grilled (a frying pan with little oil) or boiled (do not using a fryer) | 32 (82.1) | 7 (17.9) | 20 (51.3) | 19 (48.7) | -.14 | -.39 – .09 | 27 (96.4) | 1 (3.6) | 22 (78.6) | 6 (21.4) | -.06 | -.18 – .05 |
| 9. When I eat cereals (pasta, rice, quinoa, couscous), I always eat whole grains | 53 (25.6) | 154 (74.4) | 28 (13.5) | 179 (86.5) | .29^***^ | .15 – .44 | 41 (20.2) | 162 (79.8) | 37 (18.2) | 166 (81.1) | .23^***^ | .08 – .40 |
| 10. I eat a serving of natural or roasted nuts without salt at least 3 times a week | 60 (29.1) | 146 (70.9) | 30 (14.6) | 176 (85.4) | .17^**^ | .04 – .31 | 41 (20.3) | 161 (79.7) | 25 (12.4) | 177 (87.6) | .24^***^ | .09 – .41 |
| 11. At home, virgin olive oil (dark green) is used instead of sunflower oil (yellow) | 18 (90.0) | 2 (10.0) | 15 (75.0) | 5 (25.0) | -.16 | -.35 – .01 | 28 (87.5) | 4 (12.5) | 30 (93.8) | 2 (6.3) | -.09 | -.18 – .00 |
| 12. I drink commercial soft drinks, juices and/or shakes one or more times during the week | 144 (69.6) | 63 (30.4) | 158 (76.3) | 49 (23.7) | .47^*^ | .34 – .62 | 146 (71.9) | 57 (28.1) | 125 (61.6) | 78 (38.4) | .33^***^ | .20 – .46 |
| 13. When I eat a portion of dairy products, they are always natural (milk, yoghurt without sugar, fresh cheese) or minimally processed | 150 (73.2) | 55 (26.8) | 138 (67.3) | 67 (32.7) | .21^*^ | .07 – .35 | 144 (70.9) | 59 (29.1) | 123 (60.6) | 80 (39.4) | .21^**^ | .08 – .35 |
| 14. When I have breakfast, I eat pastries, cookies, juices, smoothies or processed products | 117 (56.5) | 90 (43.5) | 124 (59.9) | 83 (40.1) | .43^***^ | .31 – .56 | 123 (60.6) | 80 (39.4) | 106 (52.2) | 97 (47.8) | .51^***^ | .40 – .63 |
| 15. When I eat breakfast, I eat unprocessed or minimally processed foods (fruit, nuts, eggs, or whole wheat bread) | 80 (38.6) | 127 (61.4) | 56 (27.1) | 151 (72.9) | .37^***^ | .24 – .50 | 51 (25.1) | 152 (74.9) | 72 (35.5) | 131 (64.5) | .39^***^ | .26 – .52 |
| 16. I eat industrial pastries (sweets, cookies, snacks, or chocolate) and sweets (crisps, worms, candies, or jellies) several times a week | 117 (56.5) | 90 (43.5) | 142 (68.6) | 65 (31.4) | .42^***^ | .30 – .54 | 148 (72.9) | 55 (27.1) | 151 (74.4) | 52 (25.6) | .53^***^ | .40 – .66 |
| KIDMED index score | | | | | | | | | | | | |
| Poor (≤3) | 25 (23.4) | | 58 (107) | | -.03 | -.16 – .08 | 33 (40.2) | | 45 (54.9) | | .04 | -.13 – .23 |
| Average (4–7) | 55 (51.4) | | 45 (42.1) | |  |  | 41 (50.0) | | 35 (42.7) | |  |  |
| Good (≥8) | 27 (25.2) | | 4 (3.70) | |  |  | 8 (9.8) | | 2 (2.4) | |  |  |

**Supplemental Table 7.** *KIDMED and 7-day dietary record values of the items as a function of sex*

| KIDMED 2.0 | Girls (n = 234) | | | | | Cohen’s  Kappa | 95% CI | Boys (n = 185) | | | | Cohen’s  Kappa | 95% CI |
| --- | --- | --- | --- | --- | --- | --- | --- | --- | --- | --- | --- | --- | --- |
|  | Baseline test | | 7-day Dietary Record | | |  |  | Baseline test | | 7-day Dietary Record | |  |  |
|  | Yes (%) | No (%) | | Yes (%) | No (%) |  |  | Yes (%) | No (%) | Yes (%) | No (%) |  |  |
| 1. I eat two or more servings of fruit a day | 115 (50.0) | 115 (50.0) | | 116 (50.4) | 114 (49.6) | .35 | .23-.47 | 97 (53.9) | 83 (46.1) | 92 (51.1) | 88 (48.9) | .38*** | .24 – .52 |
| 2. I eat one or more servings of vegetables and/or raw or cooked vegetables per day | 112 (48.7) | 118 (51.3) | | 89 (38.7) | 141 (61.3) | .27*** | .15 – .39 | 82 (45.6) | 98 (54.4) | 59 (32.2) | 122 (67.8) | .27*** | .13 – .41 |
| 3. I eat one portion at lunch and another at dinner of legumes, meat (chicken, turkey or rabbit, or other lean meats), fish and/or eggs a day | 190 (82.6) | 40 (17.4) | | 123 (53.5) | 107 (46.5) | .06 | -.04 – .16 | 147 (81.7) | 33 (18.3) | 85 (47.2) | 95 (52.8) | .09 | -.02 – .20 |
| 4. More than half of the food I eat is of plant origin (fruits, vegetables, legumes, nuts, potatoes, whole grains) | 149 (64.8) | 81 (35.2) | | 113 (49.1) | 117 (50.9) | .10 | -.02 – .22 | 112 (62.2) | 68 (37.8) | 72 (40.0) | 108 (60.0) | .09 | -.05 – .23 |
| 5. When I eat lean meat, eggs and/or fish, they are usually fresh and minimally processed | 191 (83.0) | 39 (17.0) | | 159 (69.1) | 71 (30.9) | .07 | -.06 – .20 | 135 (75.0) | 45 (25.0) | 119 (66.1) | 61 (33.9) | .20* | .05 – .35 |
| 6. I eat precooked food or fast-food such as pizzas and hamburgers one or more times a week | 120 (52.2) | 110 (47.8) | | 180 (78.3) | 50 (21.7) | .20*** | .09 – .31 | 100 (55.6) | 80 (44.4) | 145 (80.6) | 35 (19.4) | .13* | .00 – .26 |
| 7. I eat three or more servings of legumes (chickpeas, white beans, lentils, peas) a week | 213 (92.6) | 17 (7.4) | | 194 (84.3) | 36 (15.7) | .31*** | .14 – .48 | 151 (83.9) | 29 (16.1) | 142 (78.9) | 38 (21.1) | .32*** | .15 – .49 |
| 8. At home, food is usually cooked in the oven, grilled (a frying pan with little oil) or boiled (do not using a fryer) | 31 (91.2) | 3 (8.8) | | 21 (61.8) | 13 (38.2) | -.17 | -.34 – .00 | 28 (84.8) | 5 (15.2) | 21 (63.6) | 12 (36.5) | .03 | -.27 – .32 |
| 9. When I eat cereals (pasta, rice, quinoa, couscous), I always eat whole grains | 42 (18.3) | 188 (81.7) | | 36 (15.7) | 194 (84.3) | .26*** | .11– .41 | 52 (28.9) | 128 (71.1) | 29 (16.1) | 151 (83.9) | .27*** | .12– .42 |
| 10. I eat a serving of natural or roasted nuts without salt at least 3 times a week | 55 (23.9) | 175 (76.1) | | 29 (12.6) | 201 (87.4) | .20** | .06 – .39 | 46 (25.8) | 132 (74.2) | 26 (14.6) | 152 (85.4) | .21** | .05 – .37 |
| 11. At home, virgin olive oil (dark green) is used instead of sunflower oil (yellow) | 25 (89.3) | 3 (10.7) | | 25 (89.9) | 10 (10.7) | -.12 | -.22 – -.02 | 21 (87.5) | 3 (12.5) | 20 (83.3) | 4 (16.7) | -.17 | -.29 – .05 |
| 12. I drink commercial soft drinks, juices and/or shakes one or more times during the week | 153 (66.5) | 77 (33.5) | | 160 (69.6) | 70 (30.4) | .33*** | -.20 – .46 | 137 (76.1) | 43 (23.9) | 123 (68.3) | 57 (31.7) | .12 | -.03 – .27 |
| 13. When I eat a portion of dairy products, they are always natural (milk, yoghurt without sugar, fresh cheese) or minimally processed | 164 (71.3) | 66 (28.7) | | 143 (62.2) | 87 (37.8) | .29*** | .16 – .42 | 130 (73.0) | 48 (27.0) | 118 (66.3) | 60 (33.7) | .10 | -.05 – .25 |
| 14. When I have breakfast, I eat pastries, cookies, juices, smoothies or processed products | 127 (55.2) | 103 (44.8) | | 126 (54.8) | 104 (45.2) | .55*** | .44 – .66 | 113 (62.8) | 67 (37.2) | 104 (57.8) | 76 (42.2) | .36*** | -.22 – .50 |
| 15. When I eat breakfast, I eat unprocessed or minimally processed foods (fruit, nuts, eggs, or whole wheat bread) | 78 (33.9) | 152 (66.1) | | 75 (32.6) | 155 (67.4) | .44*** | .32 – .56 | 53 (29.4) | 127 (70.6) | 53 (29.4) | 127 (70.6) | .28*** | .12 – .44 |
| 16. I eat industrial pastries (sweets, cookies, snacks, or chocolate) and sweets (crisps, worms, candies, or jellies) several times a week | 154 (67.0) | 76 (33.0) | | 164 (71.3) | 66 (28.7) | .53*** | .41 – .65 | 111 (61.7) | 69 (38.3) | 129 (71.7) | 51 (28.3) | .41*** | .27 – .41 |
| KIDMED index score |  | |  | |  |  |  |  |  |  |  |  |  |
| Poor (≤3) | 24 (23.5) | | 52 (51.0) | | | .06 | -.07 – .19 | 34 (39.1) | | 51 (58.6) | | -.09*** | -.24 – .05 |
| Average (4–7) | 61 (59.8) | | 56 (45.1) | | |  |  | 35 (40.2) | | 34 (39.1) | |  |  |
| Good (≥8) | 17 (16.7) | | 4 (3.9) | | |  |  | 18 (20.7) | | 2 (2.3) | |  |  |

**Supplemental Table 8.** *KIDMED 2.0 and 7-day Dietary Record Values of the Items as a function of sex*

| KIDMED 2.0 | Children (*n* = 207) | | | | Cohen’s  Kappa | 95% CI | Adolescents (*n* = 211) | | | | Cohen’s  Kappa | 95% CI | |
| --- | --- | --- | --- | --- | --- | --- | --- | --- | --- | --- | --- | --- | --- |
|  | Baseline test | | Re-test after 2 weeks | |  |  | Baseline test | | Re-test after 2 weeks | |  |  |  |
|  | Yes (%) | No (%) | Yes (%) | No (%) |  |  | Yes (%) | No (%) | Yes (%) | No (%) |  |  |  |
| 1. I eat two or more servings of fruit a day | 121 (58.5) | 86 (41.5) | 121 (58.5) | 86 (41.5) | .64^***^ | .90 – 1.10 | 93 (44.1) | 118 (55.9) | 89 (42.2) | 122 (57.8) | .61^***^ | .49 – .73 | |
| 2. I eat one or more servings of vegetables and/or raw or cooked vegetables per day | 112 (54.1) | 95 (45.9) | 104 (50.2) | 103 (49.8) | .52^***^ | .20 – .44 | 82 (38.9) | 129 (61.1) | 78 (37.0) | 133 (63.0) | .48^***^ | .36 – .60 | |
| 3. I eat one portion at lunch and another at dinner of legumes, meat (chicken, turkey or rabbit, or other lean meats), fish and/or eggs a day | 174 (84.1) | 33 (15.9) | 161 (77.8) | 46 (22.2) | .46^***^ | -.12 – .20 | 169 (80.1) | 42 (19.9) | 154 (73.0) | 57 (27.0) | .28^***^ | .14 – .42 | |
| 4. More than half of the food I eat is of plant origin (fruits, vegetables, legumes, nuts, potatoes, whole grains) | 139 (67.1.7) | 68 (32.9) | 130 (62.8) | 77 (37.2) | .48^***^ | .13 – .37 | 127 (60.2) | 84 (39.8) | 117 (55.5) | 94 (44.5) | .32^***^ | .18 – .46 | |
| 5. When I eat lean meat, eggs and/or fish, they are usually fresh and minimally processed | 165 (79.7) | 42 (20.3) | 172 (83.1) | 35 (16.9) | .54^***^ | .11 – .43 | 166 (78.7) | 45 (21.3) | 168 (79.6) | 43 (20.4) | .45^***^ | .33 – .57 | |
| 6. I eat precooked food or fast-food such as pizzas and hamburgers one or more times a week | 97 (46.9) | 110 (53.1) | 113 (54.6) | 94 (45.4) | .60^***^ | -.10 – .14 | 128 (60.7) | 83 (39.3) | 128 (60.7) | 83 (39.3) | .44^***^ | .32 – .56 | |
| 7. I eat three or more servings of legumes (chickpeas, white beans, lentils, peas) a week | 185 (89.4) | 22 (10.6) | 177 (85.5) | 30 (14.5) | .61^***^ | .00 – .20 | 186 (88.2) | 25 (11.8) | 181 (85.8) | 30 (14.2) | .48^***^ | .30 – .66 | |
| 8. At home, food is usually cooked in the oven, grilled (a frying pan with little oil) or boiled (do not using a fryer) | 181 (87.9) | 25 (12.1) | 185 (89.8) | 21 (10.2) | .22^***^ | .40 – .80 | 183 (86.7) | 28 (13.3) | 179 (84.8) | 32 (15.2) | .34^***^ | .16 – .52 | |
| 9. When I eat cereals (pasta, rice, quinoa, couscous), I always eat whole grains | 54 (261) | 153 (73.9) | 62 (30.0) | 145 (70.0) | .57^***^ | .12 – .36 | 42 (19.9) | 169 (80.1) | 59 (28.0) | 152 (72.0) | .39^***^ | .25 – .53 | |
| 10. I eat a serving of natural or roasted nuts without salt at least 3 times a week | 60 (29.0) | 147 (71.0) | 62 (30.0) | 145 (70.0) | .61^***^ | .74 – .98 | 41 (19.5) | 169 (80.5) | 54 (25.7) | 156 (74.3) | .45^***^ | .31 – .59 | |
| 11. At home, virgin olive oil (dark green) is used instead of sunflower oil (yellow) | 187 (90.3) | 20 (9.7) | 181 (87.4) | 26 (12.6) | .51^***^ | .06 – .46 | 174 (82.5) | 37 (17.5) | 179 (84.8) | 32 (15.2) | .50^***^ | .34 – .66 | |
| 12. I drink commercial soft drinks, juices and/or shakes one or more times during the week | 144 (69.6) | 63 (30.4) | 136 (65.7) | 71 (34.3) | .63^***^ | .11 – .35 | 149 (70.6) | 62 (29.4) | 140 (66.4) | 71 (33.6) | .57^***^ | .45 – .69 | |
| 13. When I eat a portion of dairy products, they are always natural (milk, yoghurt without sugar, fresh cheese) or minimally processed | 151 (72.9) | 56 (27.1) | 158 (76.3) | 49 (23.7) | .58^***^ | .16 – .44 | 149 (70.6) | 62 (29.4) | 151 (71.6) | 60 (28.4) | .56^**^ | .44 – .68 | |
| 14. When I have breakfast, I eat pastries, cookies, juices, smoothies or processed products | 116 (56.0) | 91 (44.0) | 125 (60.4) | 82 (39.6) | .59^***^ | .09 – .33 | 128 (60.7) | 83 (39.3) | 128 (60.7) | 83 (39.3) | .66^***^ | .56 – .76 | |
| 15. When I eat breakfast, I eat unprocessed or minimally processed foods (fruit, nuts, eggs, or whole wheat bread) | 81 (39.1) | 126 (60.9) | 72 (34.8) | 135 (65.2) | .53^***^ | .11 – .35 | 52 (24.6) | 159 (75.4) | 64 (30.3) | 147 (69.7) | .57^***^ | .45 – .69 | |
| 16. I eat industrial pastries (sweets, cookies, snacks, or chocolate) and sweets (crisps, worms, candies, or jellies) several times a week | 117 (56.5) | 90 (43.5) | 132 (63.8) | 75 (36.2) | .55^***^ | -.08 – .16 | 154 (73.0) | 57 (27.0) | 157 (74.4) | 54 (25.6) | .55^***^ | .41 – .69 | |
| KIDMED index score | | | | | | | | | | | | |  |
| Poor (≤3) | 52 (25.0) | | 63 (30.3) | | .52^***^ | .42 – .62 | 75 (35.5) | | 81 (38.4) | | .44^***^ | -.13 – .23 | |
| Average (4–7) | 106 (51.0) | | 100 (48.1) | |  |  | 117 (55.5) | | 97 (46.0) | |  |  |  |
| Good (≥8) | 50 (24.0) | | 45 (21.6) | |  |  | 19 (9.0) | | 33 (15.6) | |  |  |  |

**
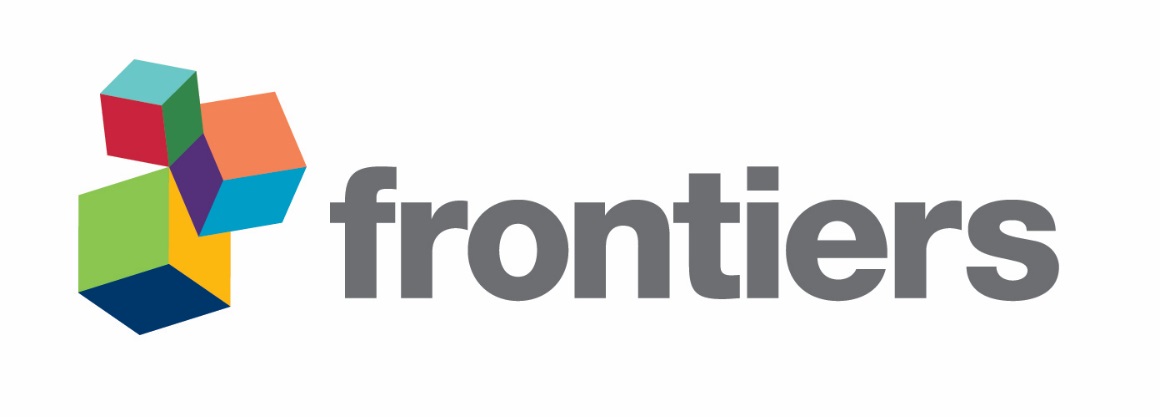
**
